# Health related quality of life of children with calcaneal apophysitis: child & parent perceptions

**DOI:** 10.1186/s12955-016-0497-4

**Published:** 2016-06-24

**Authors:** Alicia M. James, Cylie M. Williams, Terry P. Haines

**Affiliations:** Peninsula Health Service, Frankston, Australia; Monash University, Peninsula Campus, Frankston, Australia; Monash Health, Cheltenham, Australia

**Keywords:** Calcaneal apophysitis, Quality of life, Patient reported outcome measure, OxAFQ-C

## Abstract

**Background:**

Children with a clinical diagnosis of calcaneal apophysitis reportedly experience impaired physical ability. Patient reported outcome assessments measure the level of conditional specific interference in everyday life. The aim of this study was to assess and compare the child and parent perceptions of health related quality of life (QOL) associated with calcaneal apophysitis.

**Methods:**

This is a longitudinal repeated measure study nested within a randomized comparative effectiveness trial. Children who had symptoms of calcaneal apophysitis were recruited from local advertising and from the caseload of podiatrists within the health setting (Australia). The Oxford Ankle Foot Questionnaire for Children (OxAFQ-C) was completed at baseline, 1, 2, 6 and 12 month time points by both child and parent.

**Results:**

A total of 133 children were recruited and 124 participated in the study with 101 completing the OxAFQ-C at all five time points. The inter-rater reliability between the child and parent for the physical domain ranged between poor (0.06) to good (0.77) agreement, and the footwear domain ranged between poor (0.09) to good (0.66) across the time points. Both the school and emotional domains had moderate (0.46) to good (0.77) agreement.

**Conclusion:**

Children with calcaneal apophysitis have differing perceptions of health related QOL impact compared to their parents. Parents initially reported greater impact than their child however there was convergence of agreement over the follow-up period. These findings suggest understanding the impact from both child and parent perspective is imperative during treatment.

**Trial registration:**

Trial Number: ACTRN12609000696291.

## Background

Calcaneal apophysitis (Sever’s Disease) is a condition causing pain at the posterior aspect of the heel. It may present in children between the ages of 8 to 15 years [1, 2] when the apophysis of the heel is open [3, 4]. The pain is often reported as worse during and post activity and may result in limping or toe walking (to avoid ground contact) on the affected limb [5–7]. Calcaneal apophysitis is diagnosed in the absence of an injury or inflammatory medical condition and with the presentation of pain on medial and lateral compression at the posterior aspect of the heel) [8]. Children may experience pain with calcaneal apophysitis for extended periods of time before seeking treatment [9] even though calcaneal apophysitis is a self-limiting condition. Children have also reported physical, social and school impacts of calcaneal apophysitis [9] yet little is know about how parents perceive the impact of pain.

Patient reported outcome measures enable patients to describe the impact of a health condition on their quality of life (QOL) and assist clinicians to determine outcomes of treatment. Children are able to report the impact of the health condition on their quality of life and there are an increasing number of paediatric specific patient reported outcome measures. A parent proxy report of health related QOL also enables a global understanding of condition impact. Younger children may be cognitive immature, have limited social experience and dependency, therefore collecting the parent perspective is equally important to rate some aspects of their child’s QOL [10]. Older children who have the ability to read and comprehend the impact of their health on their life are ideally placed to report this to health care providers. However health care utilisation is reliant on the parent for booking, bringing the child to the appointment and assisting with any treatment regime there quality of life instruments measuring child and parent perspectives give health care providers a rounded insight into the impact of a condition [11–13].

The aim of this study was to describe and compare child and parent proxy reports of health related QOL relating to calcaneal apophysitis.

## Method

### Design

This was a longitudinal, repeated measures study nested within a randomised comparative effectiveness trial. All participants received treatment as part of the trial protocol [14]. The primary outcome of the trial was change in health related QOL between the four treatment groups. The treatment modalities included in shoe devices (orthoses or heel lifts) and footwear (current footwear/new footwear) [14]. This present study utilised outcome measurement data collected from the children and their parent at each time point. Ethics approval was obtained from Monash Health Human Research Ethics Committee (HREC 09271B) and Peninsula Health Research Ethics Committee (HREC/09/PH/65).

### Participants

The participants were children between the ages of 8–14 years who were diagnosed with calcaneal apophysitis. The diagnosis of calcaneal apophysitis was given based on history, clinical assessment and pain elicited from placing compressive force at the medial and lateral aspects of the calcaneus [8, 15]. Children were excluded from the study if they had a diagnosis or clinical signs of infective, reactive or rheumatoid arthritis, history of tumour or fracture of the foot or leg within the last 12 months or a Foot Posture Index-6 (FPI–6) equal to or less than −1 (supinated foot type). An FPI-6 equal to, or less than −1 was contraindicated to the orthotic treatment arm within the trial [14].

### Measures

Demographic data were collected from each child including age, gender, pain duration, pain laterality, height (cm) and weight (Kg). Health related QOL was collected at each point with the Oxford Ankle Foot Questionnaire for Children (OxAFQ-C) [16]. The OxAFQ-C is a patient reported outcome measure specifically developed as a child and parent proxy report of the impact of foot and ankle function and is designed for children aged between 5 and 16 years. This tool has been used to measure the foot and ankle impact of a variety of medical conditions known to cause lower limb changes including juvenile arthritis, benign joint hypermobility, Apert’s syndrome and ankle sprains [17]. It has face validity, test retest reliability and responsiveness to change in three of the four domains [16]:

1. Physical (6 items, Cronbach’s alpha = 0.92, parent-child intraclass correlation coefficient (ICC) = 0.72)
2. School and play (4 items, Cronbach’s alpha = 0.89, parent-child ICC = 0.73)
3. Emotional (4 items, Cronbach’s alpha = 0.86, parent- child ICC = 0.72)
4. Footwear (single item)

### Procedure

Recruitment advertising was sent to medical and allied health clinicians from two metropolitan health services (Monash Health and Peninsula Health), local allied health and general practitioners working in private settings. Further advertising was undertaken at local sporting clubs and school newsletters in the south eastern suburbs of Melbourne, Australia. Children participating within the study provided verbal assent and the parents provided signed consent.

**Fig. 1:**
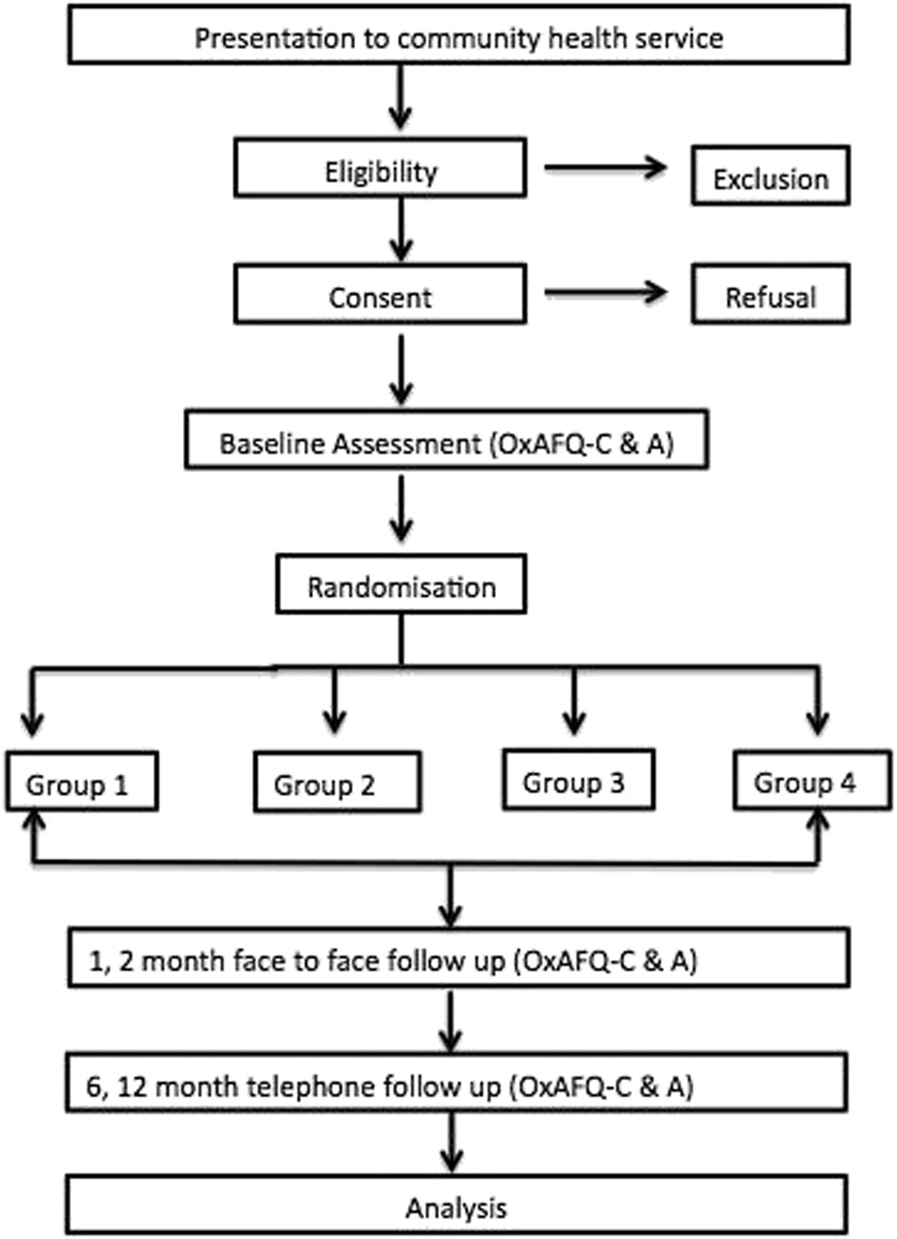
Consort Flow Chart

The demographic data were collected at the initial appointment. The parent and child separately completed the OxAFQ-C at initial presentation, the 1, 2, 6 and 12 month follow-up points (Fig. 1). The initial (124 participants), one (124 participants) and 2 month (118 participants) assessments were face to face appointments, with the parent and child completing the questionnaire by sitting separately in the treatment room so they were not able to observe each others answers. All participants were phoned at six (106 participants) and 12-month time (101 participants) point and asked to ensure they were out of hearing distance from each other while answering the OxAFQ-C questions.. In the case of non-attendance at the face-to-face appointment (2 month, *n* = 2), the OxAFQ-C was either posted by mail or a phone consultation provided for questionnaire completion with instructions for individual completion to minimize bias.

### Analysis

Analyses were conducted using Stata 13 [18]. The demographic data were summarised with means and standard deviations and OxAFQ-C summarized with medians and interquartile ranges.

The sample size for the trial was calculated based on the Oxford ankle foot questionnaire as the primary outcome measure. Previous use found that the minimum clinically important change was seven points with a maximum standard deviation of six points in any of the domains. A total of *n* = 108 was calculated to have a >90 % power to detect a significant difference of seven points, assuming a correlation between assessment points within individual participants was *r* = 0.7. To account for possible 15 % dropout, a total of 124 participants were recruited. The intraclass correlation coefficient (ICC) (Model 2 (2)) analysis was undertaken to compare the inter rater reliability between the child and parent proxy report. An intraclass correlation coefficient of <0.21 was considered to indicate poor, 021–0.40 fair agreement; 0.41 to 0.60, moderate agreement; 0.61 to 0.80, good agreement; and 0.81 to 1.00, excellent agreement [19]. Criterion validity between parent and child for the domains of the Oxford Foot Ankle Questionnaire was explored with the Bland Altman plot and the mean difference.

## Results

A total of 133 children and their parents responded to the recruitment advertisement. Participants were excluded due an FPI-6 > ^-^1 (*n* = 4), suspected diagnosis of Juvenile Idiopathic Arthritis (*n* = 1), making up pain to avoid school activity (*n* = 1) and resolved pain prior to initial assessment (*n* = 3). The remaining 124 participants consented to participate in the study (Table 1).

**Table 1:** Participant characteristics at initial presentation

Participant characteristics	Mean (SD) or n (%)
Age (year)	10.88 (1.48)
Male	72 (58)
Height (cm)	146.56 (10.37)
Weight (Kg)	41.91 (9.11)
BMI (Kg/m^2^)	19.33 (3.07)
Pain Duration (months)	10.80 (7.32)
Bilateral heel pain	106 (85 %)
Unilateral heel pain	
*Left heel*	16 (13 %)
*Right heel*	2 (2 %)

Table 2 provides summative OxAFQ-C domain data across all the time points and the level of agreement between child and parent for the four domains of the OxAFQ-C. The ICC of agreement between the child and parent for the physical domain varied from poor (0.06) to good (0.77) agreement across the five time points. Both the school and emotional domains had moderate (0.46) to good (0.77) agreement across the five time points. Child and parent agreement was observed to improve over the course of the study for the physical, emotion and social domains. The agreement within the footwear domain was noted to fluctuate over the 12 month period with the lowest level of agreement seen at the 2-month time point (0.09).

**Table 2:** Oxford ankle foot questionnaire summative data for child and parent-proxy responses

Domains	Child responses	Parent-proxy responses
	N, Median (IQR)	N, Median (IQR)
Physical domain - Baseline	124, 45.83 (35.42, 58.33)	124, 41.66 (33.33, 58.33)
Physical domain - 1 month	124, 66.66 (54.16, 75.00)	124, 66.66 (54.16, 91.66)
Physical domain - 2 months	120, 83.33 (62.50, 91.66)	120, 79.16 (66.66, 87.50)
Physical domain - 6 months	106, 79.16 (66.66, 91.66)	106, 91.66 (75.00, 100)
Physical domain - 12 months	101, 83.33 (75.00, 91.66)	101, 91.66 (70.83, 100)
School and play domain- Baseline	124, 75.00 (56.25, 87.50)	124, 75.00 (62.50, 87.50)
School and play domain - 1 months	124, 87.50 (75.00, 100)	124, 87.50 (75.00, 100)
School and play domain - 2 months	120, 100.00 (81.25, 100)	120, 100.00 (81.25, 100)
School and play domain - 6 months	106, 100.00 (93.75, 100)	106, 100.00 (87.50, 100)
School and play domain 12 months	101, 100.00 (100, 100)	101, 100.00 (93.75, 100)
Emotional domain- Baseline	124, 87.50 (75.00, 100)	124, 93.75 (81.25, 100)
Emotional domain - 1 month	124, 93.75 (81.25, 100)	124, 100.00 (87.50, 100)
Emotional domain - 2 months	120, 100.00 (93.75, 100)	120, 100.00 (93.75, 100)
Emotional domain - 6 months	106, 100.00 (93.75, 100)	106, 100.00 (100, 100)
Emotional domain - 12 months	101, 100.00 (93.75, 100)	101, 100.00 (100, 100)
Footwear domain- Baseline	124, 75.00(50.00, 100)	124, 75.00 (50.00, 100)
Footwear domain - 1 months	124, 75.00 (50.00, 100)	124, 50.00 (50.00, 75.00)
Footwear domain - 2 months	120, 100.00 (75.00, 100)	120, 50.00 (25.00, 75.00)
Footwear domain - 6 months	106, 75.00 (75.00, 100)	106, 100.00 (75.00, 100.)
Footwear domain – 12 months	101, 100.00 (75.00, 100)	101, 100.00 (75.00, 100)

**Fig. 2:**
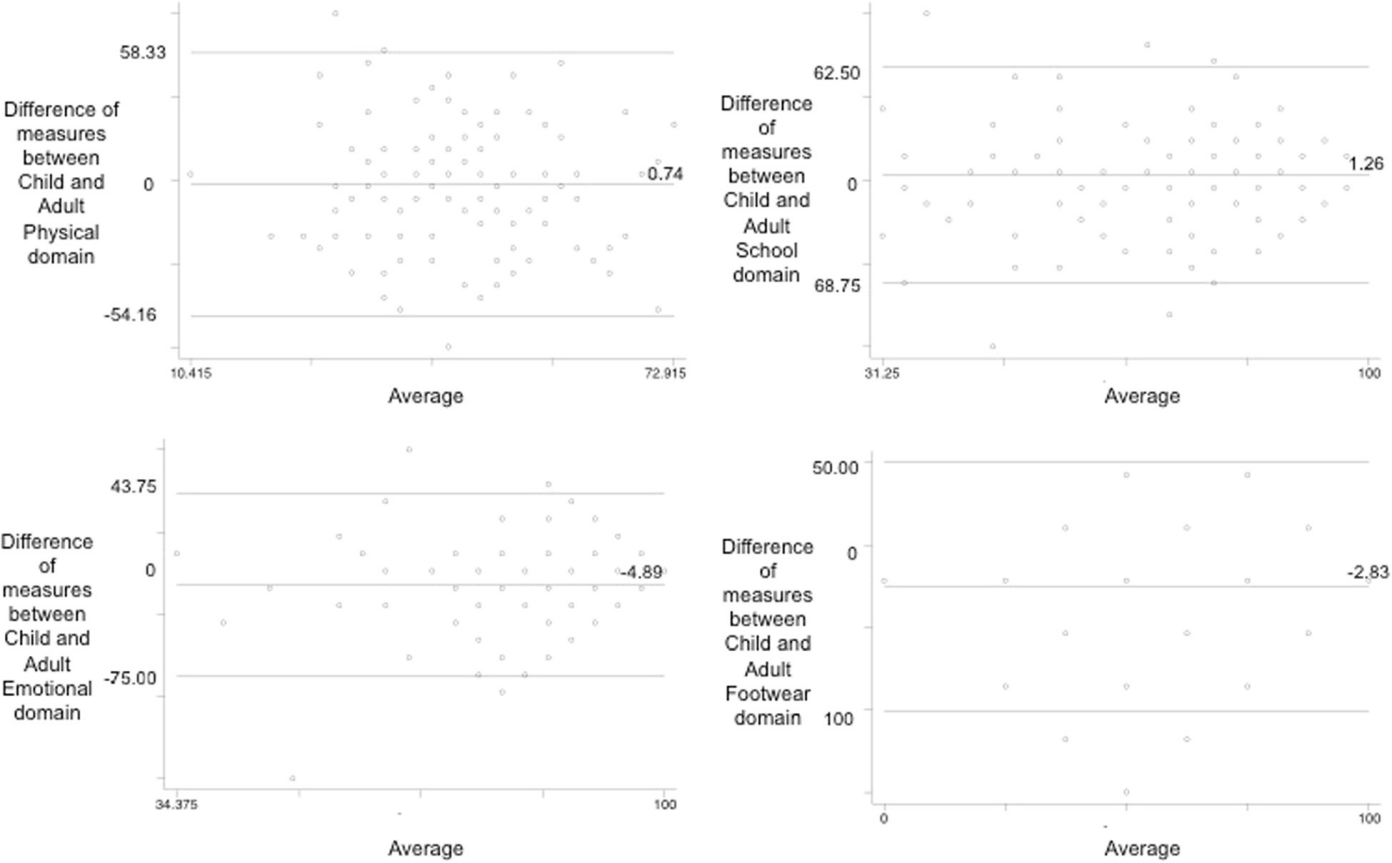
Bland Altman plot baseline- Oxford ankle foot questionaire

**Fig. 3:**
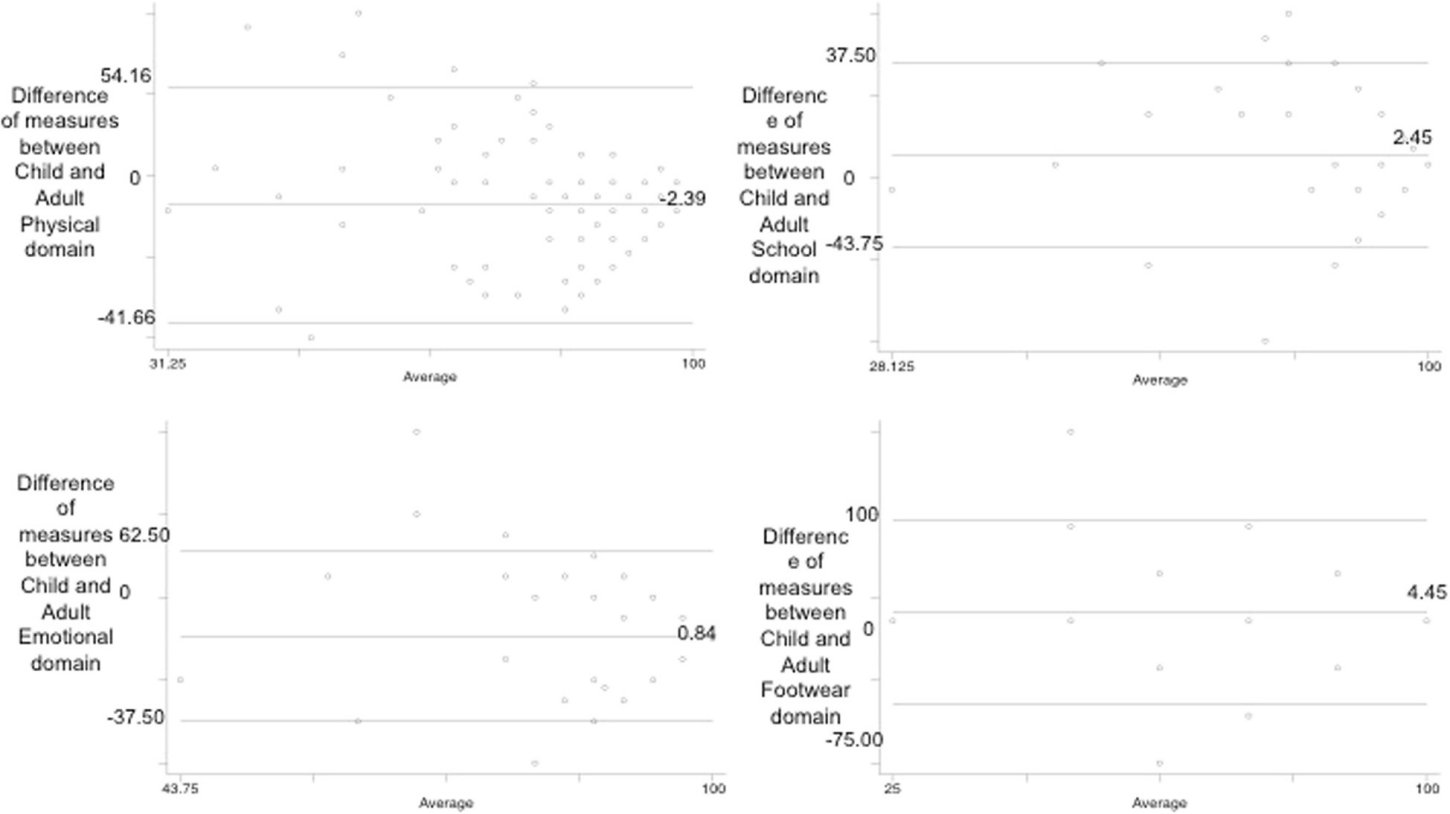
Bland Altman plot 12 months- Oxford ankle foot questionaire

Agreement between the child and parent across all domains was explored using a Bland Altman plot at baseline and 12 month time points for all four domains (Figs. 2 and 3). The 95 % CI for limits of agreement within each domain (Table 3) displayed a relatively large spread of score difference between child and parent assessments at every time point.

**Table 3:** Levels of agreement/inter-rater agreement between child and parent

Domain	Time period	95 % limits of agreement	Mean difference	95 % CI of mean difference	Difference range child minus parent	ICC	ICC 95 % CI
Physical	Baseline	−42.80, 43.34	0.74	−3.20, 4.67	−54.15, 58.33	0.06	−0.34, 0.34
	1 month	−39.96, 33.80	−3.05	−6.32, 0.20	−58.33, 70.83	0.67	0.53, 0.77
	2 month	−27.76, 31.42	1.84	−0.83, 4.52	−41.66, 37.50	0.77	0.67, 0.84
	6 month	−38.73, 27.64	−5.54	−8.74, −2.34	−58.33, 37.50	0.71	0.55, 0.81
	12 month	−37.28, 32.49	−2.39	−5.84, 1.05	−41.66, 54.16	0.70	0.56, 0.80
School	Baseline	−43.88, 41.34	−1.27	−5.03, 2.51	−68.75, 62.50	0.62	0.46, 0.74
	1 month	−32.21, 35.06	1.41	−1.56, 4.39	−56.25, 50.00	0.73	0.61, 0.81
	2 month	−24.33, 26.11	0.88	−1.39, 3.16	−31.25, 37.50	0.70	0.56, 0.79
	6 month	−31.42, 29.34	−1.04	−3.97, 1.88	−96.00, 37.50	0.59	0.40, 0.72
	12 month	−20.52, 25.43	2.45	−0.18, 4.72	−43.75, 37.50	0.77	0.65, 0.84
Emotional	Baseline	−37.80, 28.03	−4.89	−7.81, −1.96	−75.00, 43.75	0.62	0.45, 0.74
	1 month	−29.60, 23.60	−2.97	−5.33, −0.62	−50.00, 31.25	0.74	0.63, 0.82
	2 month	−21.94, 21.84	−0.05	−2.03, 1.92	−25.00, 50.00	0.47	0.23, 0.64
	6 month	−21.85, 19.85	−1.00	−3.01, 1.01	−43.75, 31.25	0.47	0.22, 0.64
	12 month	−24.70, 26.39	0.84	−1.67, 3.36	−37.50, 62.50	0.61	0.42, 0.74
Footwear	Baseline	−61.67, 56.03	−2.83	−8.05, 2.41	−100.00, 50.00	0.58	0.41–0.71
	1 month	−52.52, 75.69	11.58	5.27, 16.90	−75.00, 75.00	0.15	−0.17–0.38
	2 month	−39.20, 98.78	29.79	23.55, 36.02	−50.00, 75.00	0.09	−0.16–0.25
	6 month	−51.89, 47.18	−2.35	−7.12, 2.41	−75.00, 100	0.66	0.50–0.77

## Discussion

This study demonstrated that the children with calcaneal apophysitis within this trial reported substantially different health related QOL impact than their parents when using the OxAFQ-C. This difference was noted to be mostly a random rather than a systematic difference, as a majority of the domain scores were not significantly different between child and parent scores. These findings mean that parent scores should not be used, as proxy scores to represent what children would otherwise have said. This does not mean that parents should not be asked these questions, as parents are an important stakeholder in the child/parent diad or triad.

There was poorer agreement in the physical domain at baseline or when the impact of pain was at it’s most prevalent, however there was good agreement in the three other domains at this time point. The difference in agreement between child and parent for the physical domain at baseline may be the reason why there was an average pain duration of 10.8 months prior to seeking medical intervention. Agreement in the physical domain improved in line with the improvement of symptoms over the 12 months. The school and emotional domains were observed to have fluctuating levels of agreement across the 12 month period but there was consistent moderate to good agreement over the 12 months. The footwear domain (single item) was noted to have significantly different agreement across the 12 month period in comparison to the other domains. It was observed the level of agreement between child and parent deteriorated from moderate to poor post intervention. The parents reported calcaneal apophysitis had a higher impact on footwear choice particularly at the first one and two months of the trial. One of the interventions within this trial was the provision of athletic footwear and this disparity may be due to frequent parental prompts to wear the intervention footwear.

The OxAFQ-C has previously been found to have good parent and child agreement within a convenience sample of children presenting to an orthopaedic clinic. Conditions presenting to the clinic included congenital clubfoot, planovalgus flat foot (hypermobility), acute pain or trauma and cerebral palsy [20]. This higher level of agreement for the physical domain was significantly different to what was determined in the child and parent proxy reports for children with calcaneal apophysitis. Children with calcaneal apophysitis often have intermittent and activity related pain rather than constant discomfort. This intermittent nature may be the reason the children report significant differences in impact given that some children may be in pain at the consultation or relying on pain recall from the time the child was last active. This was a limitation of the study, as not all children had similar baseline pain levels. The disagreement between child and parent proxy reports specifically indicates this difference in view of pain impact. Low agreement also highlights the importance of gathering data from both child and parent perspective due to different but valid information. A parent of an older child may also vary in awareness, sensitivity or tolerance of their child’s intermittent heel pain and this may be reflected in the score. Many other studies investigating treatment options for children with calcaneal apophysitis have recruited from junior sporting clubs or sports academies. This present study recruited from the general population and included children with low to moderate levels of activity participation. Therefore a parent or child who has similar activity participation goals may have different levels of agreement that those observed from the general population. In spite of this, the parents reported greater impact on health related QOL in this instance than the child. One could postulate that a parent’s observation of limping or reduced participation in sport may be reflected in these scores but the child sees this limitation as so temporary that they rate less impact.

This study has been the first to examine the longitudinal health related QOL impact of calcaneal apophysitis from both the child and parent’s perspective. Previous studies have utilized QOL tools that are not lower limb and/or musculoskeletal specific, and have not been validated for the age group examined [21]. For example, the child and parent-proxy version of the OxAFQ-C was utilized with children who have symptomatic flexible flat foot and compared to their peers with typical flat foot, the OxAFQ-C was also found to elicit specific impact rather than the generic PedsQL™ 4.0 [22].

Researcher should consider investigating the minimally important clinical difference that will of the OxAFQ-C to detect a change during treatment. Further research is also required to explore the quality of life and disability impact of calcaneal apophysitis in comparison to other lower limb conditions. This research would allow greater insight to the level of disability experienced by children when experiencing lower limb pathologies.

## Conclusion

Children with calcaneal apophysitis have a differing perception of health related QOL impact compared to their parent’s perception. The parents reported a greater impact than the child initially however there was convergence of agreement over time following treatment. These findings suggest calcaneal apophysitis has a considerable impact on the health related QOL understanding the impact from both child and parent perspective is imperative during treatment. Further research examining and comparing the scores of the OxAFQ-C related to other lower limb conditions will determine the magnitude of the impact.
